# Plant protection, the Cinderella of the one health strategy?

**DOI:** 10.1016/j.onehlt.2025.101080

**Published:** 2025-05-18

**Authors:** Giacomo Lorenzini, Cristina Nali

**Affiliations:** Department of Agriculture, Food and Environment of the University of Pisa, 56124 Pisa, Italy

**Keywords:** Food security, Food safety, Ecosystem services, Antimicrobial resistance, Agenda 2030, Phytopathology

## Abstract

Plant and microbiome health are fundamental to life on Earth, human well-being, and planetary health, yet their importance is often underestimated. Human and animal health depend on plant health for food security (nutritious food production, economic growth), food safety (contamination risks), ecosystem services (air quality, mental health benefits), and mitigating antimicrobial resistance (from agricultural antibiotic use). Plant health specialists are crucial for plant health, food security, and achieving the Sustainable Development Goals within a One Health framework. Plant diseases and pests can significantly impact food production, economic stability, and public health. To address these challenges, we need to integrate plant health into the One Health framework. This requires collaboration between plant scientists, veterinarians, human health experts, and environmental scientists. By working together, we can develop more sustainable and holistic approaches to agriculture and public health.

## 1 Introduction

**Fig. 1:**
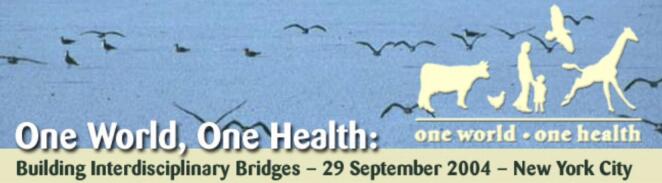
The official logo of the “One World, One Health” event: Please note the absence of any reference to the plant world.

The “One Health” concept, while not recent, has become crucial for tackling modern health challenges. It emphasizes the interconnectedness of human, animal, and environmental health, requiring interdisciplinary collaboration. Originating from the “One Medicine” idea (1984) which focused on human and animal health, “One Health” evolved in 2004 at a symposium emphasizing a broader, interdisciplinary approach to global health threats, though notably excluding plant health from the discussion [1,2]. The outcome of the symposium, known as the “Manhattan Principles,” included 12 recommendations for creating a more holistic approach to preventing epidemic and epizootic diseases, and for maintaining ecosystem integrity to benefit humans, domestic animals, and the biodiversity that underpins all life [3]. Plants and plant health were conspicuously absent from these principles, which concentrated almost entirely on human and animal health. The conference logo (Fig. 1) also omitted any reference to the plant world.

**Fig. 2:**
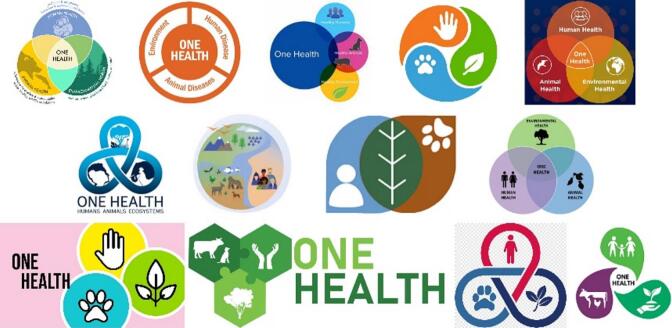
A selection of logos designed to promote events and initiatives related to One Health. Notice how, graphically, the role of plants (and their health) is equal to humans and animals. “One health”, represented by the triad “Healthy People, Healthy Animals, Healthy Environments”, is an approach in which multiple sectors work together to achieve better public health outcomes.

Facing climate change and biodiversity loss, the need to update the Manhattan Principles became urgent. Consequently, the German Federal Foreign Office and the Wildlife Conservation Society held the “One Planet, One Health, One Future” conference (October 25, 2019), bringing together nearly 200 participants from 47 countries. Leading up to the conference, 12 experts formulated the “Berlin Principles on One Health,” which expand upon the Manhattan Principles by integrating ecosystem health and addressing key issues such as pathogen spillover, climate change, and antimicrobial resistance [4]. The One Health concept, now widely recognized, has spurred numerous scientific initiatives and events. Dozens of scientific journals now cover these topics. While the equality of human, animal, and plant health is formally acknowledged, as evidenced by various logos (Fig. 2) and initiatives, the active participation of plant health specialists remains limited. They are often underrepresented in key roles such as board memberships and keynote speaker position at major international events. Plant pathology studies are significantly underrepresented in One Health journals, with a few exceptions [e.g. [5,6]].

The 2024 G7 and G20 declarations prioritize human and animal health in their application of One Health, as evidenced by a word count showing plants mentioned only 12 times (AMR 89, ecosystems five). This focus, likely due to the declarations being issued by health authorities, reinforces the perception that One Health is predominantly about human health [7]. However, the One Health Commission's definition explicitly includes plant health as a crucial component of a collaborative, multisectoral, and transdisciplinary approach to optimal health and well-being. This holistic approach emphasizes the interconnectedness of people, animals, plants, and their shared environment [8].

In light of this scenario, the purpose of this article is to elucidate the supplementary benefits derived from a holistic consideration of human, animal, plant, and ecosystem health, with the goal of definitively establishing plant health as an intrinsic element of the One Health framework. Drawing upon an analysis of specialist literature, the present article (a “position paper” authored by two seasoned plant pathologists active in sustainable crop protection and plant/environmental pollutant interactions) explores the crucial role of plant health within the One Health strategy, focusing on key areas such as food security and safety, toxic metabolites produced by plant pathogens affecting humans and animals, chemical contaminants transferred by plants into the food chain, zoonotic pathogen contamination of plant-based foods, ecosystem services and plant health, and antimicrobial resistance.

## 2 The role of plants in One Health

Plants are essential for human life, providing basic necessities and contributing to individual well-being, environmental sustainability, and planetary health [9]. They support ecosystems, regulate cycles, and mitigate climate change. Plants face numerous abiotic and biotic environmental challenges [10], impacting ecosystems and agriculture. Plant health is crucial for plant, human, animal, and environmental health, as well as the global economy. Globalization and climate change exacerbate plant-enemy relationships, with increased trade facilitating pest spread and climate change weakening plant resilience [11].

**Fig. 3:**
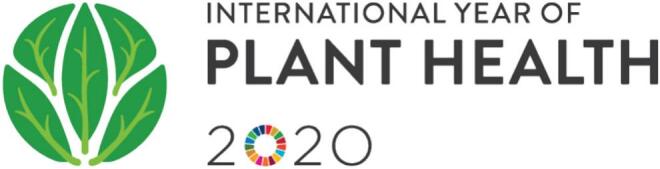
The official logo of the International Year of Plant Health established by the United Nations in the year 2020.

Modern plant pathology, a discipline with deep historical roots, emerged from one of history's darkest chapters: the Great Famine in Ireland. In the 1840s, the arrival of a virulent strain of *Phytophthora infestans* devastated potato crops, leading to widespread famine and mass migration [12]. This tragedy underscored the devastating impact of plant diseases and spurred scientific inquiry into their causes. Pioneers like Berkeley and de Bary recognized the fungal nature of the blight pathogen, a groundbreaking discovery that predated Pasteur's germ theory [13]. This realization laid the foundation for modern plant pathology and highlighted the critical role of microorganisms in disease development. Today, plant health is a global concern. The United Nations has designated 2020 as the *International Year of Plant Health* (Fig. 3) and May 12th as the *International Day of Plant Health*. These initiatives aim to raise awareness of the importance of plant health in addressing global challenges such as hunger, poverty, and environmental degradation. By understanding and managing plant diseases, we can safeguard our food security, protect ecosystems, and contribute to a sustainable future.

### 2.1 Food security: a cornerstone for sustainable development

Food security, as defined by the 1996 World Food Summit [14], means that all people, at all times, have access to safe and nutritious food to meet their dietary needs for a healthy life. This involves balancing food availability, access, supply, and utilization. Achieving food security is essential for the 2030 Agenda's Sustainable Development Goals (SDGs), especially SDG 1 (*Ending Poverty*) and SDG 2 (*Zero Hunger*) [15]. Addressing poverty requires boosting farmer incomes (through pest control, trade, and jobs), while ending hunger and malnutrition depends on improving food availability, access, and affordability. Food security is linked to other SDGs, like good health (SDG 3) and strong institutions (SDG 16). Family farming is crucial for food production, rural economies, and social well-being. Protecting plant health and promoting agricultural trade are key to food security and supporting the livelihoods of billions reliant on family farms. Cash crops, grown for profit, are vital for many economies, especially in developing countries. Examples include cocoa, coffee, fiber crops, sugarcane, rubber, tea, and tobacco. While profitable, they don't always contribute directly to food security. Food crops, like cereals, pulses, roots, and tubers, are primarily for domestic consumption [16]. Bananas, for example, serve as both food staple and cash crop in many low-income countries [17]. Plant diseases and pests threaten both cash and food crops, reducing yields, increasing costs, and driving up food prices.

### 2.2 Standing examples of catastrophic plant diseases

The 1970 Southern Corn Leaf Blight epidemic in the US powerfully illustrates the dangers of crop genetic uniformity. This devastating event, caused by the fungus *Cochliobolus heterostrophus* (*Drechlera maydis*), demonstrates the critical role of crop diversity in disease mitigation and food security [18,19]. The widespread use of corn hybrids reliant on Texas male-sterile cytoplasm, a trait simplifying seed production, created a monoculture highly vulnerable to the emerging “race T" strain of the fungus. Combined with favorable weather, the outbreak caused widespread crop failure and economic hardship, highlighting the risks of monoculture and the necessity of genetic diversity in agriculture [20]. Numerous plant diseases have the potential to devastate crops and threaten the livelihoods of communities worldwide [21]. One such disease is Tropical Race 4 (TR4), a soil-borne fungal strain that causes Fusarium wilt in bananas [22]. Fusarium wilt, caused by the fungus *Fusarium oxysporum* f. sp. *cubense*, can severely damage banana plants by blocking their vascular systems. The highly contagious TR4 strain spreads through contaminated soil, water, and plant material, and it is nearly impossible to eradicate once established. This pathogen poses a significant threat to global banana production, especially to the dominant Cavendish cultivar. Originating in Asia in the 1970s, TR4 has since spread to Africa and, more recently, Latin America [23]. This is particularly concerning for many low-income countries where bananas are a staple food and a significant cash crop. The disease can lead to significant yield losses, increased production costs, and economic hardship for small-scale farmers. The date palm (*Phoenix dactylifera*) is economically, ecologically, and culturally vital to many North African and Middle Eastern countries [24]. It provides diverse products and holds deep cultural significance. However, Fusarium wilt, caused by *Fusarium oxysporum* f. sp. *albedinis*, has decimated millions of trees in Morocco and Algeria, impacting high-quality cultivars and displacing farmers [25]. Developing resistant cultivars is a complex but essential process for the industry's survival. Coffee rust, caused by the fungus *Hemileia vastatrix*, significantly threatens global coffee production, particularly *Coffea arabica*, causing severe leaf damage and yield losses [26,27]. Originating in 19th-century Sri Lanka, it has spread to coffee-producing regions worldwide, jeopardizing smallholder farmers and economies. Difficult to control and currently incurable, coffee rust, despite mitigation efforts, persists as a challenge with far-reaching economic and social consequences for coffee-dependent communities [28].

These and other tragic examples highlight the vulnerability of regions heavily reliant on a single crop or a few species. When these crops are devastated by disease, the consequences can be severe, particularly in developing countries. These nations often face rapid population growth, widespread poverty, and limited resources for agricultural research and extension services. In such contexts, the failure of a major crop can have catastrophic consequences for food security and livelihoods.

### 2.3 Food safety: protecting public health

Food safety is crucial for public health, involving practices for handling, preparing, and storing food to prevent foodborne illnesses. These illnesses, caused by harmful biological or chemical contaminants, can range from mild to life-threatening [29]. Globalization and climate change complicate food safety due to complex supply chains and potential impacts on food production and distribution. Prioritizing food safety measures at every stage, including hygiene, storage, and cooking, is essential. Plant diseases not only decrease yields but also diminish fruit quality and nutritional value [30]. Additionally, food safety can be compromised by physical, biological, and chemical hazards. Physical hazards include foreign objects like glass, metal, or plastic, as well as naturally occurring substances like fish bones [31]. Food can be contaminated by biological hazards (bacteria, viruses, parasites) and chemical hazards (e.g., pesticide residues), both posing risks to human health. Effective plant protection, especially through integrated pest management, is crucial to minimize pesticide use, maintain crop health, and ensure food safety.

### 2.4 Plant pathogens and human/animal health

While plant and animal pathogens usually target different hosts, some plant pathogens can infect humans and animals, though this is rare [32]. Differences in plant and animal physiology, along with host defenses like stomach acid, make cross-kingdom infection challenging. Plant pathogens' specialized adaptations often limit their ability to infect animals and humans. However, emerging evidence suggests some plant pathogens can cause disease in humans and animals, highlighting the potential risk of such cross-kingdom infections. So, for instance, plant viruses are not considered to present any potential pathogenic threats to humans or other vertebrates, although some plant viruses may replicate within the bodies of insect hosts that are acting as transmission vectors [33]. While the gastric route is generally not a concern, clinical isolates of plant pathogenic microorganisms are primarily found in immunocompromised, post-surgical, or post-traumatic patients, with a few exceptions. This includes some *Agrobacterium tumefaciens* (now *Rhizobium radiobacter*) and *Erwinia* isolates. These bacterial phytopathogens are typically considered opportunistic, lacking human/animal specificity. As expected, no host-specific virulence factors have been identified in these clinical phytopathogens. Similarly, *Alternaria infectoria*, a fungus causing blossom blight in guayule, caused phaeohyphomycosis in a post-renal transplant patient [34]. The human eye, exposed to the environment and possessing a unique structure, is susceptible to various infectious diseases, including fungal keratitis. This condition, often caused by fungi like *Fusarium*, *Alternaria*, and *Aspergillus*, all of them able to live as saprophyte/parasite on plants [35] and leads to corneal opacity and even blindness. The increased use of contact lenses further heightenes the risk of fungal keratitis [35]. While these fungi typically inhabit plants, they can opportunistically infect humans, particularly those with weakened immune systems or corneal injuries [36]. In addition to eye infections, *Fusarium oxysporum* has been associated with nail infections and invasive fungal infections in immunocompromised individuals [37]. In healthy individuals, *Fusarium* species can cause fungal sinusitis [38].

### 2.5 Metabolites produced by plant pathogens toxic to humans/animals

Although plant pathogens rarely infect humans and animals directly, they can produce harmful toxins called ‘mycotoxins’. These fungal metabolites, produced by *Aspergillus*, *Penicillium*, and *Fusarium* species, can contaminate food crops like cereals and grains, posing significant health risks, including liver damage, cancer, and neurological disorders. Mycotoxins can enter the human food chain through contaminated plant-based foods or animal products (meat, milk, eggs) [39]. Even with food safety regulations and temperate climates, mycotoxins remain a persistent problem in developed countries, requiring continuous monitoring, control, and management [40]. The economic ramifications of mycotoxin-contaminated animal feed include reductions in feed intake, feed refusal, diminished feed conversion efficiency, impaired body weight gain, increased disease incidence (attributable to immune suppression), and compromised reproductive capacities [41,42]. Mitigating mycotoxin risks requires strict food safety regulations and effective monitoring. Good agricultural practices and proper storage minimize fungal growth and toxin production. Many countries, guided by organizations like the FAO, WHO, and EU [43], have regulations limiting mycotoxin concentrations in food and feed.

Ergot alkaloids, produced by *Claviceps* fungi (especially *C. purpurea*), contaminate cereals like rye, wheat, triticale, and oats. These fungi form dark structures called sclerotia, which contain high concentrations of the toxins [44,45]. Consuming contaminated feed causes serious health problems in livestock by disrupting various physiological processes. In humans, ergot alkaloid poisoning can lead to vomiting, diarrhea, muscle spasm, and hallucinations [46]. Historically, ergot poisoning caused severe illness and death. While strict legal limits on ergot alkaloids in processed cereals [47] have largely eliminated human ergotism, it remains a significant veterinary concern, particularly for cattle, horses, sheep, pigs, and poultry [48,49]. Effective crop management and food safety measures are crucial for mitigation. Celery, like many other vegetables, is susceptible to fungal infections, including those caused by *Sclerotinia sclerotiorum*. As a defense mechanism, the plant produces furocoumarin compounds. These molecules can cause a skin condition known as ‘phytophotodermatitis’ in individuals who handle the plant, leading to painful blisters on the skin [50].

### 2.6 Chemical contaminants introduced by plants in the food chain

**Fig. 4:**
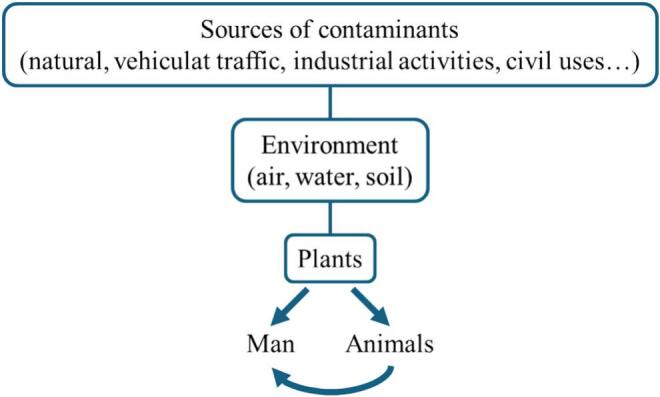
Chemical contamination pathways and the food safety concept. Food contaminants in food and feed may be authorized (e.g. pesticides) or unauthorized (e.g. heavy metals).

Food plants, interacting with air, water, and soil, are susceptible to chemical contamination from industrial emissions, human activities, or natural sources, impacting their composition and safety (Fig. 4). Plants can absorb/adsorb pollutants, becoming a critical link in the pollution pathway to humans. Monitoring and regulating chemical contaminant levels in food is crucial for safety. Appropriate agricultural practices, food processing standards, and regulations can minimize these risks.

Persistent contaminant uptake by edible plants and their accumulation in the food chain poses a significant threat to human and animal health, causing problems from mild poisoning to severe organ damage [51]. Heavy metals (HMs), dense metallic elements, are primarily released by human activities like mining, industry, and fossil fuel/waste combustion. HMs are categorized as *essential* (required for biological functions like growth and metabolism, e.g., copper, iron, manganese, cobalt, zinc) and *nonessential* (no known biological role, toxic even at low levels, e.g., cadmium, lead, mercury, chromium, aluminum) [52]. Plants absorb HMs from soil (roots) and air (leaves/deposition), which can then disrupt the function of essential biomolecules like proteins and enzymes [53]. The most widespread HM contaminants in food are lead, cadmium, arsenic, and chromium. The *Codex Alimentarius* has established limits for these metals in various food products [54].

Pesticides, both mineral and organic, are widely used in agriculture to protect crops. After application, they degrade, leaving residues in food depending on the pesticide, application method, and crop. Post-harvest treatments can also affect residue levels, which are regulated to minimize consumer exposure [55]. However, enforcement is often lax in developing countries. The European Food Safety Authority's 2022 monitoring found 96.3 % of samples compliant with maximum residue limits (MRLs), with 3.7 % exceeding them (2.2 % non-compliant after accounting for uncertainty) [56].

### 2.7 Plant food contaminated with zoonotic pathogens

Foodborne illnesses from bacteria, viruses, and parasites are a major global health concern. Many pathogens contaminate raw food [57], including fruits, vegetables, and sprouts exposed to contaminated manure or water. These contaminated items can then enter the food chain. Human and animal pathogens can colonize plants and their roots. Improper storage and processing further contribute to contamination. Ready-to-eat produce is especially vulnerable. Despite the misconception that plants play a minor role, fresh produce causes numerous foodborne outbreaks. A prime example is Shiga toxin-producing *Escherchia coli* (STEC), especially the O157:H7 serovar, responsible for thousands of annual illnesses in the US [58]. A severe *E. coli* O157:H7 outbreak linked to pre-packaged spinach caused numerous hospitalizations and deaths, tracing back to a single farm contaminated by irrigation water or deer [59]. STEC produces Shiga toxins, damaging the intestines, kidneys, and brain, causing abdominal cramps, bloody diarrhea, and kidney damage, sometimes leading to neurological and heart problems. A 2011 O104:H4 outbreak in Europe, primarily Germany, caused nearly 4000 infections, over 900 cases of HUS, and 54 deaths [60]. This outbreak was linked to contaminated sprouted seeds from a single organic producer in Germany, sourced to fenugreek seeds from Egypt [61]. *Salmonella* is a leading cause of foodborne illness, traditionally thought to spread mainly through cross-contamination [62]. However, recent research has shown that certain *Salmonella* strains can directly colonize and multiply on plants, posing a significant risk to public health [63]. *Salmonella* has evolved mechanisms to evade plant defenses, allowing it to survive and replicate within plant tissues. This ability to colonize plants can lead to contamination of food products, even in the absence of cross-contamination [64]. A notable example of a *Salmonella* outbreak linked to contaminated produce is the 2009 outbreak of *S. typhimurium* associated with peanut butter. This outbreak affected over 600 people and resulted in nine deaths. A single facility run by a major player in the peanut butter industry was identified as the source of contamination [65]. Human norovirus and hepatitis A virus are the leading viral causes of foodborne illness worldwide, often transmitted through fresh and frozen berries like strawberries and raspberries. Contamination can occur via contact with contaminated surfaces, water, or hands. These viruses survive freezing and other harsh conditions, retaining their infectivity, and the risk of infection is high due to raw or minimal processing of many berries [66,67].

## 3 Ecosystem services and plant health

Ecosystem services are the benefits humans derive from nature, categorized into four types [68]: *Supporting* (nutrient cycling, soil formation, primary production); *Regulating* (climate regulation, air/water purification, flood control); *Provisioning* (food, water, timber, fuel, medicinal plants); and *Cultural* (recreation, aesthetics, spiritual well-being).

**Fig. 5:**
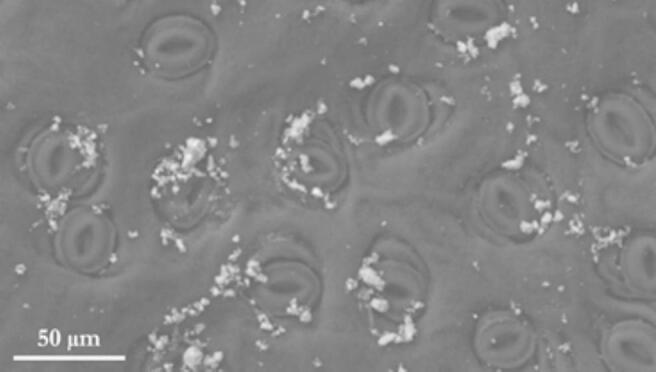
Particulate matter deposited on the abaxial leaf surface of *Pittosporum tobira*.

Urban green spaces (parks, gardens, street trees) provide numerous ecosystem services benefiting human health [69] including air quality improvement (pollutant absorption); climate regulation (urban heat island mitigation); mental health benefits (stress, anxiety, depression reduction); and physical health benefits (physical activity promotion, chronic disease risk reduction). Protecting and restoring ecosystems ensures these essential services for future generations. With half the world's population now living in cities (and projected to reach 70 % by mid-century, adding 2.5 billion people, mainly in Asia and Africa), the urban heat island effect will intensify, exacerbating the dangers of climate change-driven heatwaves [[70], [71], [72], [73]]. These heatwaves, particularly devastating in Europe (as the 2003 heatwave demonstrated [74]), claim thousands of lives, especially among the elderly, women, and disadvantaged. Urban greening, including parks and trees, can mitigate this urban heat and offer psychological benefits, improving human health and well-being [75]. Urban forests also play a crucial role in combating air pollution, a major global health concern linked to millions of deaths annually [76,77]. Plants remove gaseous and particulate air pollutants through their stomata and leaf cuticles, respectively [78] (Fig. 5). This process is effective for both outdoor pollutants, such as ozone [79] and indoor pollutants, including formaldehyde, benzene, and xylene [80].

In the Madrid region (Spain) the role of El Pardo peri-urban forest (16,000 ha surface) in modifying ozone concentrations has been analyzed using air quality models. The results show that El Pardo forest constitutes an efficient sink of ozone since (virtually) removing this green area significantly modified ozone metrics increasing levels over the modified area and over down-wind surrounding areas [81].

Invasive plant pests and pathogens significantly disrupt ecosystem services. The Emerald Ash Borer (*Agrilus planipennis*, EAB), native to Asia, has decimated ash trees in North America, with ecological consequences including reduced water regulation, soil erosion, carbon sequestration, and altered forest succession [82,83]. In urban settings, EAB infestations can worsen the urban heat island effect and increase energy consumption and air pollution-related health issues. Ash tree loss creates canopy gaps, impacting understory vegetation, nutrient cycling, and forest succession, potentially favoring invasive species. The borer attack has been linked to over 6000 respiratory-related and 15,000 cardiovascular deaths in 15 US states [84]. Phytophthora dieback, caused by the invasive water mold *Phytophthora cinnamomi* [85], poses a major threat to biodiversity in Southwest Australia [86]. Likely introduced via contaminated soil or plant material, it infects and kills diverse plant species, and its spread is facilitated by human activities [87]. Phytophthora dieback prevents infected plants from developing new shoots, flowers, fruit, and seeds, ultimately leading to their death and ecosystem breakdown [88]. With no known cure, it can deplete a species' seed bank and cause local extinctions, resulting in reduced biodiversity, biomass, habitat loss, food scarcity for native animals, increased weed invasion, and bare soil [88]. Described as a “biological bulldozer” [89], its impact may be comparable to highly disruptive invasive plants [90].

*Xylella fastidiosa*, a devastating bacterial plant pathogen spread primarily by insects like the meadow spittlebug, has caused significant global damage [91]. While historically confined to the Americas, it was detected in southern Italy in 2013, devastating olive trees and other plants [92]. With no cure, infection is usually fatal [93]. In Italy, nearly 21 million olive trees across 54,000 ha have been lost [94], representing an irreplaceable loss of cultural heritage and vital ecosystem services, particularly the monumental olive trees of Salento [94]. This loss has disrupted local ecology, increasing soil temperature, erosion, and evaporation; decreasing CO_2_ sequestration and regional precipitation; heightening flood and wildfire risk; and negatively impacting olive production, ornamental resources, recreation, and ecotourism [94].

While ecosystems provide numerous benefits (ecosystem services), they can also have negative impacts on human well-being, known as “ecosystem disservices” [95]. These can manifest as: (1) *Direct Impact* (e.g., pollen allergens, falling trees, dangerous wildlife); (2) *Diminished Ecosystem Services* (e.g., crop pests reducing yields); and (3) *Loss of Supporting Services* (e.g., wildfires degrading soil) [96]. Urban green spaces, despite their benefits, can harbor allergenic plants [97], and urban trees, while beneficial, can pose risks from falling branches/trees (exacerbated by climate change), sometimes leading to unnecessary fear and tree removal [98]. Urban trees also present challenges related to air pollution. While many volatile organic compounds (VOCs) contributing to ozone (O_3_) formation are human-caused, trees also emit biogenic VOCs (BVOCs) like isoprene and monoterpenes, which can contribute to O_3_ production, especially during summer's peak photochemical activity and BVOC emissions [[99], [100], [101]]. Other disservices include infrastructure damage, vector-borne disease harborage, blocked sunlight, increased energy use, maintenance costs, perceived unattractiveness (of unmanaged areas), and potential issues with urban wildlife (bats, rats, wolves) [102]. Balancing the benefits and risks of urban trees requires careful planning, maintenance, and an understanding of their complex ecological role. Understanding both the positive and negative aspects of ecosystems is crucial for sustainable management and human well-being.

## 4 Antimicrobial resistance: a growing threat

Antimicrobial compounds, like antibiotics, revolutionized 20th-century medicine. However, their widespread and often indiscriminate use has led to antimicrobial resistance (AMR), a serious global health concern [103]. While AMR can occur naturally, human activities accelerate it [104]. Overuse in medicine, veterinary medicine, and agriculture creates selective pressure, allowing resistant strains to flourish. Microorganisms develop resistance through intrinsic mechanisms (lack of target site) or acquired mechanisms (genetic mutations/gene transfer), which can alter the target site or enhance the microorganism's ability to degrade/eliminate the chemical. Agricultural antimicrobial use can unintentionally select for resistant microorganisms in both target and non-target species. Unchecked AMR could cause 10 million deaths annually by 2050 [105]. Bacteria can develop AMR through two primary mechanisms: (*i*) *Genetic Mutation*: Random genetic mutations can confer resistance by altering the target site of the antibiotic or by increasing the microorganism's ability to eliminate the drug, and (*ii*) *Horizontal Gene Transfer*: Bacteria can acquire resistance genes from other bacteria through various genetic exchange processes. Antibiotics exert strong selective pressure on bacterial populations, favoring the survival and reproduction of resistant strains. Over time, these resistant strains can become dominant, leading to the widespread dissemination of AMR [106].

The consumption of food treated with antibiotics can expose the human gut microbiome to sub-therapeutic levels of these drugs. This exposure can select for antibiotic-resistant bacteria, as the low-level antibiotic pressure favors the growth of resistant strains [107,108]. Furthermore, bacteria isolated from plant-based foods often carry genes that confer resistance to a wide range of antibiotics used in human and animal medicine [109]. While the exact amount of antibiotics used in agriculture is difficult to quantify due to a lack of global monitoring, it's estimated that around 30 countries permit their use in crop protection. Although the overall amount of antibiotics used in agriculture is relatively low compared to human and veterinary medicine, it still contributes to the problem of AMR [110]. Several classes of antibiotics used to treat human and animal diseases are also employed in agriculture to combat bacterial pathogens. These include aminoglycosides, tetracyclines, and quinolones [108]. While the use of antibiotics in crop production is relatively low compared to livestock and aquaculture, representing only 0.26 % to 0.5 % of total agricultural antibiotic consumption in the USA [111], it still contributes to the overall problem of antimicrobial resistance.

Streptomycin, once used to treat tuberculosis, is now widely used in horticulture, primarily against fire blight in fruit trees. However, its agricultural use is restricted or banned in Europe due to widespread streptomycin resistance in both human and plant pathogens [112,113]. Resistance mechanisms include altered ribosome binding, reduced uptake, and enzymatic degradation. Besides fruit trees, rice is another major crop where antibiotics are recommended [114]. Animal manure can contain up to 90 % unmetabolized antibiotics [115], introducing residues into the soil and potentially selecting for resistant bacteria [116]. The rhizosphere, rich in nutrients, harbors a diverse microbial community, including bacteria with high antibiotic resistance due to horizontal gene transfer [117].

Azole fungicides, used extensively in agriculture, veterinary medicine, and human health, target a fungal cell membrane synthesis enzyme. Their widespread agricultural use has led to resistance in 30 plant pathogens across over 60 countries [118], reducing their effectiveness. Mitigation strategies include rotating fungicides and reducing applications, but cross-resistance is a concern, as resistance to agricultural azoles can confer resistance to medical azoles [119]. This agricultural use has also contributed to azole resistance in human pathogenic fungi like *Aspergillus*, *Fusarium*, and *Candida* [120]. *A. fumigatus*, sharing environments with plant pathogens, is exposed to agricultural azole selective pressure, and studies link field resistance to residual azole levels [121].

Vector-borne diseases pose a significant threat, especially to vulnerable populations. While insecticides have been vital for control, increasing insecticide resistance is undermining their effectiveness [122]. Widespread agricultural insecticide use has contributed to resistance in disease-carrying insects like mosquitoes, hindering malaria and other vector-borne disease control. Alternative and integrated vector control strategies are essential to minimize insecticide reliance and resistance development [123].

## 5 Concluding remarks

**Fig. 6:**
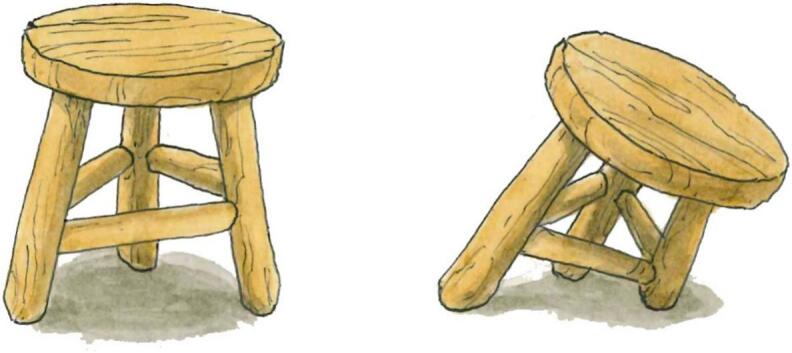
This figure illustrates the delicate balance of the One Health approach, likened to a three-legged stool. Each leg – human health, animal health, and ecosystem health – must be strong and stable to support the overall structure. A weakened leg can destabilize the entire system, compromising the health of all.

Just as a three-legged stool is most stable when all legs are of equal length (Fig. 6), the One Health concept is strongest when human health, animal health, and environmental health are equally prioritized. Unfortunately, environmental health, particularly plant health, is often overlooked.

While the One Health approach typically focuses on zoonotic diseases, incorporating plant health can strengthen the framework. By aligning with UN Sustainable Development Goals, we can emphasize ecological health and balance the need for food security with planetary boundaries. The One Health approach emphasizes the interconnectedness of human, animal, and environmental health. By adopting this holistic perspective, we can develop more inclusive and sustainable plant protection strategies. Healthy plants are essential for food security, environmental health, and human well-being. Crop diseases and pests can significantly reduce yields, leading to food shortages and malnutrition. Additionally, unhealthy plants can disrupt ecosystems, impacting biodiversity and climate regulation. Effective plant protection practices are crucial for global food security and environmental sustainability. By considering the broader implications of plant health, we can develop more sustainable and resilient agricultural systems that benefit both people and the planet. Plant health is crucial for global food security, environmental sustainability, and human well-being. Healthy plants not only provide food and nutrition but also contribute to ecosystem services like carbon sequestration and biodiversity. However, plant health is often overlooked in discussions of One Health. While the focus is typically on zoonotic diseases, the interconnectedness of plant, animal, and human health is undeniable. Adopting a One Health perspective can offer significant advantages to plant professionals, and it would be beneficial for them to be fully aware of this. The main obstacle remains the lack of coordination between sectors, as plant health involves numerous stakeholders in agriculture, farming, the environment, and health [124]. Plant diseases and pests can significantly impact food production, economic stability, and public health. To address these challenges, we need to integrate plant health into the One Health framework. This requires collaboration between plant scientists, veterinarians, human health experts, and environmental scientists. Incorporating plant protection into the One Health framework is essential for strengthening agri-food supply chain resilience. Healthy plants underpin food security, safety, and economic stability, and their protection reduces risks such as yield loss, contamination, and antimicrobial resistance. To ensure sustainable and resilient food systems, plant health must be recognized as a critical component of public and planetary health, supported by cross-sector collaboration and integrated policy action. Plant professionals should keep in mind the benefits of operating within the context of the One Health strategy and recall that the challenge is the lack of inter-sectoral coordination as plant health involves many stakeholders across the agriculture, farming, environment and health sectors. By working together, we can develop more sustainable and holistic approaches to agriculture and public health.

## CRediT authorship contribution statement

**Giacomo Lorenzini:** Writing – review & editing, Writing – original draft, Visualization, Validation, Supervision, Software, Resources, Project administration, Methodology, Investigation, Funding acquisition, Formal analysis, Data curation, Conceptualization. **Cristina Nali:** Writing – review & editing, Writing – original draft, Visualization, Validation, Supervision, Software, Resources, Project administration, Methodology, Investigation, Funding acquisition, Formal analysis, Data curation, Conceptualization.

## Funding

This study was funded by the University of Pisa.

## Declaration of competing interest

The authors declare that they have no known competing financial interests or personal relationships that could have appeared to influence the work reported in this paper.

## Data Availability

No data was used for the research described in the article.
